# Supplementary material for: Benzo(a)pyrene and Cerium Dioxide Nanoparticles in Co-Exposure Impair Human Trophoblast Cell Stress Signaling
Source: Int J Mol Sci. 2023 Mar 12;24(6):5439. doi: 10.3390/ijms24065439 (PMC10049531; doi:10.3390/ijms24065439)
Supplement: Supplementary file 1 [file ijms-24-05439-s001.zip › Figure S1. Diapositive10.pdf]

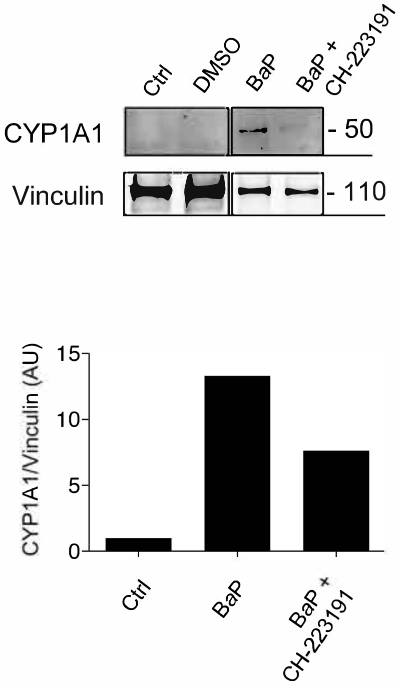

Supplementary Figure S1. Effect of AhR antagonist on CYP1A1 induction

VCT purified from term placentas were plated overnight and were pre-incubated for 1 h with AhR antagonist (3 μM) then either untreated or incubated with B*a*P (0.6 μM) for 24 h. Total protein extracts were subjected to SDS-PAGE under reducing conditions and membranes were immunoblotted with anti-CYP1A1 and anti-vinculin antibodies (the latter used as loading control). Immunoblots were quantified with an Odyssey System Imager and results are shown in the bar scale graph as ratio to the vinculin (n=1).
