# Supplementary material for: Benzo(a)pyrene and Cerium Dioxide Nanoparticles in Co-Exposure Impair Human Trophoblast Cell Stress Signaling
Source: Int J Mol Sci. 2023 Mar 12;24(6):5439. doi: 10.3390/ijms24065439 (PMC10049531; doi:10.3390/ijms24065439)
Supplement: Supplementary file 1 [file ijms-24-05439-s001.zip › Figure S2. Diapositive11.pdf]

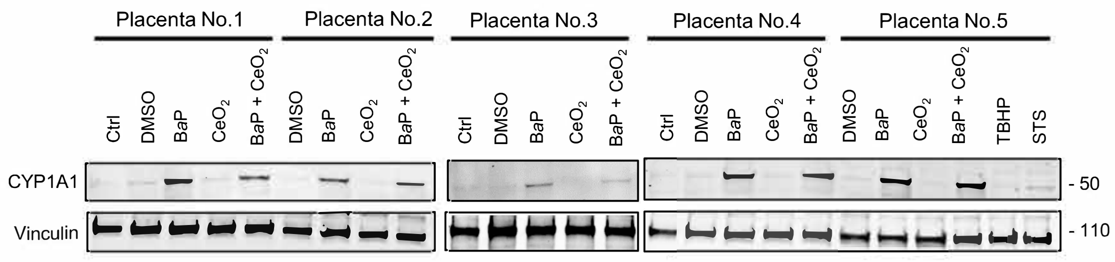

Supplementary Figure S2. Homogeneity of the cellular response of the different placentas

VCT purified from term placentas were plated overnight and were either untreated (Control), incubated with DMSO, B*a*P (0.6 μM) or CeO_2 NP (6.3 μg/cm^2) and in B*a*P and CeO_2 co-exposure for 24 h. Apoptosis controls with 0,5 μM STS and 100 μM TBHP treatments were done for 4 h and 3 h respectively. Total protein extracts were subjected to SDS-PAGE under reducing conditions and membranes were immunoblotted with anti-CYP1A1, anti-p53 and anti-vinculin antibodies (loading control).
