# Supplementary material for: Benzo(a)pyrene and Cerium Dioxide Nanoparticles in Co-Exposure Impair Human Trophoblast Cell Stress Signaling
Source: Int J Mol Sci. 2023 Mar 12;24(6):5439. doi: 10.3390/ijms24065439 (PMC10049531; doi:10.3390/ijms24065439)
Supplement: Supplementary file 1 [file ijms-24-05439-s001.zip › Figure S3. Diapositive12.pdf]

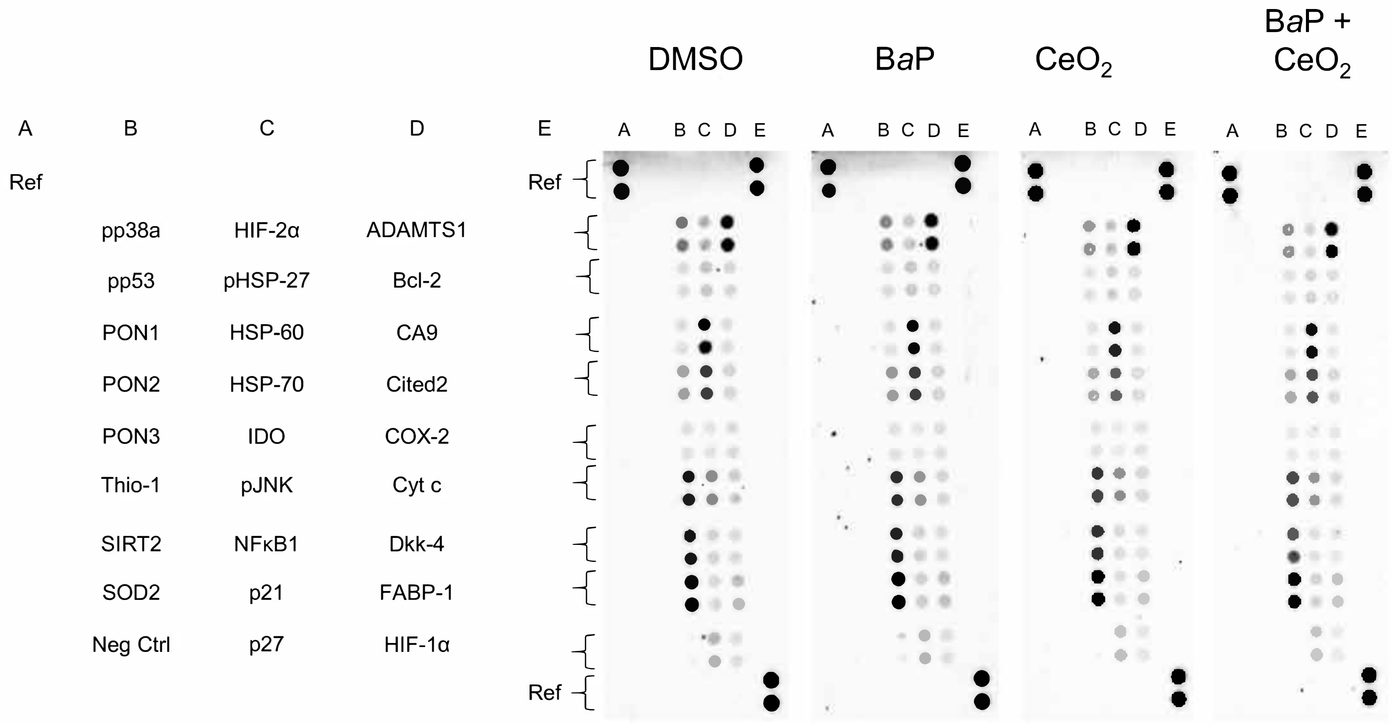

Supplementary Figure S3. Immunoblots of Cell Stress Array experiments

VCT purified from term placentas were plated overnight and were either incubated with DMSO, B*a*P (0.6 µM) or CeO_2 NP (6.3 µg/cm2) and in co-exposure for 24 h for Cell Stress Array. Immunoblots were quantified with an Odyssey System Imager (n=5). Numbers in the table represent quantification of duplicate samples for each protein as ratio to the DMSO control (arbitrary units).

Proteins	B*a*P	CeO_2	B*a*P + CeO_2	Phosphorylation sites
**ADAMTS1**	1.102	0.953	0.990	
**Bcl-2**	1.000	0.888	0.920	
**CA9**	1.019	0.874	0.780	
**Cited-2**	1.060	0.841	0.942	
**COX-2**	0.902	0.870	0.812	
**Cyt c**	0.994	1.092	0.978	
**Dkk-4**	0.987	0.809	0.795	
**FABP-1**	0.982	0.898	0.881	
**HIF-1a**	0.929	0.859	0.712	
**HIF-2a**	0.695	0.733	0.521	
**phospho-HSP27**	0.931	0.749	0.752	S78/S82
**HSP60**	0.802	0.804	0.804	
**HSP70**	0.903	0.711	0.822	
**IDO**	0.937	0.848	0.843	
**phospho-JNK Pan**	0.857	0.897	0.837	T183/Y185
**NFkB1**	1.083	0.850	0.708	
**p21**	1.414	0.889	1.303	
**p27**	1.047	0.920	0.870	
**phospho p38**	0.897	0.790	0.823	T180/Y182
**phospho p53**	1.156	0.917	0.985	S46
**PON1**	0.896	0.905	0.852	
**PON2**	1.029	0.872	0.898	
**PON3**	0.953	0.941	0.811	
**Thioredoxin-1**	0.880	0.846	0.829	
**SIRT2**	0.969	0.899	0.726	
**Mn SOD2**	1.016	0.916	0.863	

ADAMTS1, a desintegrin and metalloproteinase with thrombospondin motifs 1; Bcl-2, B cell lymphoma-2; CA9, carbonic anhydrase 9; Cited-2, Cbp/p300-interacting transactivator; COX-2, cyclooxygenase-2; Cyt c, cytochrome C; Dkk-4, dickkopf-4; FABP-1, fatty acid binding protein-1; HIF-1◎, hypoxia inducible factor 1 alpha; HIF-2◎, hypoxia inducible factor 2 alpha; pHSP27, phospho heat shock protein-27; HSP-60, heat shock protein-60; HSP70, heat shock protein-70; IDO, indoleamine 2,3-dioxygenase; pJNK, phospho c-Jun n-terminal kinase; NF◎B1, nuclear factor kappa B1; p21, cyclin-dependent kinase inhibitor 1A; p27, cyclin-dependent kinase inhibitor 1B; pp38◎, phospho-p38 alpha; pp53, phospho-p53; PON1, paraoxonase 1; PON2, ; PON3, paraoxonase 3; Thio-1, thioredoxin-1; SIRT2, sirtuin 2; SOD2, superoxide dismutase 2; Neg Ctrl, negative control.
